# Tumor Microenvironmental Dynamics in Shaping Resistance to Therapeutic Interventions in Melanoma: A Narrative Review

**DOI:** 10.3390/ph18081082

**Published:** 2025-07-22

**Authors:** Laci M. Turner, Hanna Terhaar, Victoria Jiminez, Bailey J. Anderson, Emily Grant, Nabiha Yusuf

**Affiliations:** 1 Heersink School of Medicine, University of Alabama at Birmingham, Birmingham, AL 35233, USA; 2 Department of Dermatology, University of Alabama at Birmingham, Birmingham, 1670 University Blvd., VH566A, Birmingham, AL 35294, USA

**Keywords:** tumor microenvironments, melanoma, immune checkpoint inhibitors, resistance, PD-L1, CTLA-4

## Abstract

**Background/Objectives**: This review discusses the resistance mechanisms in the tumor microenvironment (TME) of malignant melanoma that disrupt the efficacy of immune checkpoint inhibitors (ICIs). In this review, we focus on the roles of immune cells, including tumor-infiltrating lymphocytes (TILs), macrophages, dendritic cells, and other signaling pathways. We explore the interplay between innate and adaptive immunity in the TME and tumor intrinsic resistance mechanisms, such as β-catenin, which has future implications for the usage of ICIs in patients with therapy-resistant tumors. **Methods**: A total of 1052 studies were extracted from the PubMed database searching for keywords and phrases that included [melanoma AND immune checkpoint inhibitor resistance]. After a title/abstract and full-text review, 101 studies were identified that fit the inclusion/exclusion criteria. **Results**: Cancer-associated fibroblasts (CAFs), M2 macrophages, and myeloid-derived suppressor cells (MDSCs) are significant in remodeling the TME to promote melanoma growth. Melanoma resistance to ICIs is complex and involves TME alterations, tumor intrinsic factors, and immune evasion. Key components of resistance include reduced CD8+ T cell infiltration, decreased host immune response, and immunosuppressive cytokines. **Conclusions**: Predictive biomarkers and specific models are the future of individualized melanoma management and show great promise in their approach to targeted therapy production. Tumor profiling can be utilized to help predict the efficacy of ICIs, and specific biomarkers predicting therapy responses are instrumental in moving towards personalized and more efficacious medicine. As more melanoma resistance emerges, alternative and combinatorial therapy based on knowledge of existing resistance mechanisms will be needed.

## 1. Introduction

The landscape of melanoma has transformed in recent decades with the emergence of immune checkpoint inhibitors (ICIs), such as ipilimumab, nivolumab, and pembrolizumab. These therapeutic agents block immune-inhibitory receptors, such as cytotoxic T-lymphocyte-associated protein 4 (CTLA-4) and programmed cell death protein (PD-1) on activated T cells to promote host immunity against tumor cells and improve T cell functionality [1,2]. While ICIs have been shown to improve survival outcomes for nodal and distal metastatic melanoma, many patients lack clinical responses to these therapies or develop therapy resistance over time [1,2,3].

Response rates are hypothesized to be related to the presence and activity of existing T cell populations, also referred to as tumor-infiltrating lymphocytes (TILs) within the tumor microenvironment (TME) before therapy [1,2,3]. This activity can be assessed by a baseline in CD8+ T cell infiltration within the TME [1]. For instance, melanomas with a T-cell-inflamed phenotype respond better to ICIs. In contrast, melanomas with non-T cell inflamed phenotype (via preventing T-cell infiltration through activation of β-catenin, etc) have poorer responses to therapy [1]. This difference in responses is due to the significant impact of T cells, which recognize antigens on melanoma cells and initiate various immune responses [1]. 

However, the interaction between ICIs and the TME is more complex than targeting one specific pathway: intrinsic tumor signaling, such as β-catenin, can lead to ineffective immunotherapy responses in certain subsets of patients. Moreover, the immunosuppressive TME in melanoma is created and maintained via alterations in tumor cells, stromal cells, and regulatory lymphocytes that decrease host surveillance [4]. In addition to its TME, melanoma can quickly adapt and mutate its phenotype in response to external factors that threaten tumorigenesis [2,5]. By identifying specific mechanisms and markers leading to resistance against ICIs in the TME, emerging therapies can be specifically designed to decrease immune evasion and improve immunotherapy response. In this review, we aim to provide a comprehensive review of resistance mechanisms in response to ICIs and other immunotherapy approaches for melanoma and suggest ways to overcome them that may emerge as therapeutic targets in the coming years. 

## 2. Discussion

### 2.1. Overview of the TME

The TME refers to the landscape surrounding a tumor that contributes to the tumor’s growth, longevity, and resistance to various treatments and therapies [6]. TME has been a topic of interest since 1889, when Stephen Paget’s famous “soil and seed” provided an ideal representation of the interplay between a tumor and its TME [7]. The tumor is considered the “seed”, and the TME is the “soil”, providing nourishment and support for the tumor to plant its roots and grow and thrive. Therefore, the TME is an important target for immunotherapies, as the tumor cannot survive without the support system of its environment. The TME consists of both cellular and noncellular components: cellular components of the environment include stromal cells, endothelial cells, immune cells, and tumor cells.

### 2.2. Essential Components of the TME 

#### 2.2.1. Stromal Cells

Stromal cells communicate with microenvironment components by inducing cytokines and mediators via paracrine signaling to attract tumor cells and orchestrate tumor cell invasion, angiogenesis, and proliferation [8]. They decrease cancer cell sensitivity to medications by releasing growth and inflammatory factors, contributing to a dysregulated extracellular matrix (ECM). Overall, the action of stromal cells helps create an environment-mediated drug resistance (EM-DR) [9]. 

Studies on the role of stromal cells, such as fibroblasts, in the TME have identified at least two cancer-associated fibroblast (CAF) types: a myofibroblast adjacent population solely focusing on tissue remodeling and an inflammatory population specializing in immune system adaptation [10]. CAFs release interleukin-6 (IL-6), which acts through the signal transducer and activator of transcription 3 (STAT-3) pathway to activate inflammation and angiogenesis [10]. CAFs also release transforming growth factor-beta (TGF-β), which increases fibronectin synthesis to remodel the ECM, activates anti-inflammatory M2 macrophages, and drives immunosuppression. TGF-β levels have been correlated with T cell dysfunction in melanoma and have also been shown to increase expression of forkhead box P3 (FOXP3), which controls regulatory T cell (Treg) development [11,12,13]. 

Leukemia-inhibitory factor (LIF) released by the tumor promotes CAF activation and helps promote the pro-cancer response of TGF-β. CAF release of chemokine (C-X-C motif) ligand 1 and 2 (CXCL1 and CXCL2) also increases tumor invasion, with CXCL12 further increasing macrophage recruitment, and CXCL1 increasing tumor invasion via activation of matrix-metallopeptidase 1 (MMP1), also known as interstitial collagenase [10].

**Figure 1:**
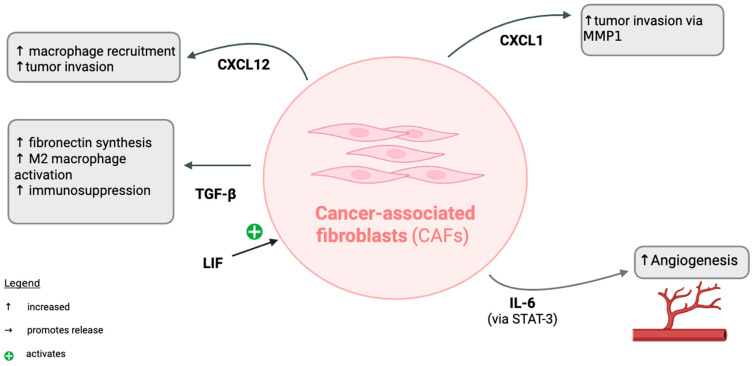
The role of CAFs in the TME. This image was created in Biorender. Turner, L. (2025) https://app.biorender.com/illustrations/6811922f1183103c4a3060df?slideId=7182ae12-d8ff-45bf-a1b0-6c8f4024ea0a. The figure depicts the roles of CAFs in promoting tumor progression and immunosuppression within the TME. CAFs contribute to macrophage recruitment and tumor invasion through CXCL12 signaling and promote tumor invasion via matrix metalloproteinase 1 (MMP1) through CXCL1. Transforming growth factor-beta (TGF-β) released by CAFs increases fibronectin synthesis, M2 macrophage activation, and immunosuppression. Leukemia inhibitory factor (LIF) activates CAFs, while CAF-derived interleukin-6 (IL-6) signaling through signal transducer and activator of transcription 3 (STAT-3) promotes angiogenesis. Symbols: ↑ indicate increased activity, → indicates promotion of release, and + indicates activation.

In summary, CAFs release various signaling molecules that support tumor growth and invasion by promoting ECM remodeling, angiogenesis, and immunosuppression, as shown below in Figure 1. 

CAFs modify the TME to support tumor growth and invasion via the release of cytokines to increase ECM remodeling, tumor invasion, and immunosuppression [10]. 

#### 2.2.2. Endothelial Cells

Endothelial cells are key players in promoting angiogenesis, forming tight junctions, undergoing endothelial to mesenchymal transition, and secreting cytokines. Angiogenesis is essential to expanding the malignant potential of the tumor by supporting its growth and hematogenous spread. Tight junction formation is a mechanism of resistance through host immune evasion, with endothelial cell tight junctions acting as a barrier against immune cells attempting to infiltrate tumor cells and allowing the tumor to survive [14]. These cells also undergo endothelial-to-mesenchymal transition to promote tumor growth by allowing intravasation and extravasation of tumor cells. Lastly, they secrete cytokines to bolster tumor immunity while hindering antitumor immune responses [14]. 

#### 2.2.3. Immune Cells

Adaptive and innate immune cells play a dynamic and essential role in the metastasis and survival of the tumor cells. Innate cells that have been studied to play an active role in the TME include macrophages, natural killer (NK) cells, dendritic cells (DCs), and neutrophils. Tumor-associated macrophages (TAMs) play a significant role in the immunological landscape and are regarded as the predominant group of tumor-infiltrating immune cells [15]. They can transform into both M1- and M2-like macrophages. M1 macrophages have anti-cancer and pro-inflammatory effects, such as phagocytosis, lysis, induction of apoptosis via cytokines, tumor necrosis factor (TNF), IL-6, IL-12, and IL-23, and the ability to enhance CD8+ T cells [15]. They are activated by toll-like receptors (TLRs) and Th1 cytokines, and induce the production of STAT1, STAT2, and ROS while decreasing expression of the immunosuppressive cytokine IL-10 [16]. Decreased IL-10 contributes to enhanced CD8+ T cell activity, allowing the M1 macrophage to carry out its pro-inflammatory effects. Although M1 macrophages are regarded as anti-tumor, cancer stem cells can still survive via M1 macrophage filtration [15]. M2 macrophages have pro-cancer and anti-inflammatory effects through their generation of TGF-β, an anti-inflammatory mediator that causes the induction of Tregs, and their production of arginase, which metabolizes L-arginine [15]. Tregs and arginase contribute to a tumor-friendly environment where immune responses are suppressed, contributing to further tumor progression and potential resistance to immunotherapy [15,17]. M2 macrophages also induce the production of STAT3 and STAT6 [16]. MicroRNAs (miRs) have been found to play a key role in macrophage differentiation. MiR-155 has been found to promote an M1 macrophage response, enhancing the pro-inflammatory response of T-cells in the context of tumor cell invasion [18]. However, miR-21 strongly promotes an M2 macrophage response that promotes tumor growth and resistance [18]. The role of miRs in tumor cell proliferation, macrophage differentiation, and chemoresistance is further discussed later in the text.

**Figure 2:**
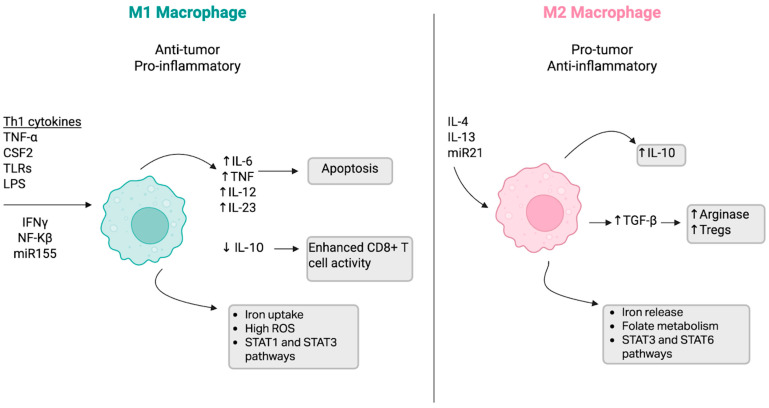
Distinct Roles and Functions of M1 and M2 Macrophages. This image was created in Biorender. Turner, L. (2025) https://app.biorender.com/illustrations/684359509b3f24b278204c4a?slideId=bf028f10-1653-44be-892b-db982d377b95. This figure shows an overview of M1 and M2 macrophage polarization and their roles in the TME. M1 macrophages (**left**) are anti-tumor and pro-inflammatory, activated by Th1 cytokines (tumor necrosis factor-alpha (TNF-α), colony-stimulating factor 2 (CSF2), toll-like receptors (TLRs), and lipopolysaccharide (LPS)), leading to interferon-gamma (IFN-γ), nuclear factor kappa B (NF-κβ), and miR155 activation. M1 macrophages increase interleukin (IL)-6, TNF, IL-12, and IL-23 while decreasing IL-10, promoting enhanced CD8+ T cell activity and apoptosis. They are associated with iron uptake, high reactive oxygen species (ROS), and STAT1 and STAT3 pathway activation. M2 macrophages (**right**) are pro-tumor and anti-inflammatory, induced by IL-4, IL-13, and miR21, leading to increased IL-10 and transforming growth factor-beta (TGF-β), which in turn enhance arginase expression and regulatory T cell (Treg) development. M2 macrophages are associated with iron release, folate metabolism, and activation of STAT3 and STAT6 pathways.

This distinction between M1 and M2 macrophages can be seen in Figure 2. 

M1 macrophages have anti-tumor and pro-inflammatory effects, while M2 macrophages have pro-tumor and anti-inflammatory effects. 

The role of NK cells in the TME is unclear. However, studies have found that the density of NK cells in the TME influences the prognosis of the associated cancer [19]. High densities of NK cells in the TME are good prognostic factors in many cancers [20]. Tumor-infiltrating DCs can identify and display tumor-associated antigens to naive T-lymphocytes, ultimately resulting in an anti-tumor T-cell response. However, the anti-tumor response of the DCs can be hijacked by the TME, altering DC phenotypes and inducing tolerogenic regulatory DCs to support tumor proliferation and advancement while promoting immunosuppression and preventing effector T cell activity [21]. Neutrophils contribute to the TME by releasing various cytokines and chemokines, such as proteases, that promote angiogenesis and degrade the extracellular environment for tumor evasion [22].

Adaptive immune cells also play a critical role in the TME. When the TME acquires Tregs, they play a pro-tumor response by suppressing anti-tumor immune responses, promoting tumor metastasis and growth [23]. T-cells in the TME can support the tumor through a variety of pro-tumor mechanisms, such as secreting a variety of proteases to break down and remodel the ECM [23]. Dysregulated T-cells in the TME have decreased effector production and upregulated expression of inhibitory receptors due to the exhaustion of overworked T-cells in chronic cancer microenvironments [24]. Cytotoxic CD8+ cells are susceptible to immunosuppression and immune escape by the TME due to various factors, such as cytokines and chemokines secreted by stromal cells, macrophages, and endothelial cells [24,25].

#### 2.2.4. Tumor Cells

The tumor cells, mentioned above as the seed of the TME, are malignant melanoma. This cancer arises from melanocytes in the epidermis of the skin. These cells are the pigment-producing cells that are derived embryologically from neural crest cells. Malignant transformation of melanocytes causes melanoma, often through exposure to UV radiation. The malignant cells interact with the surrounding TME utilizing various chemical and ligand-mediated interactions to evade host immune response and grow. Many forms of therapy-resistant melanomas have developed, the mechanisms of which will be discussed in further detail within this review.

#### 2.2.5. Non-Cellular Components

Non-cellular components of the TME include the ECM, growth factors, metabolic interactions, cytokines, and chemokines. The ECM has been studied to provide a barrier for the cancer cells, prohibiting cancer-targeting drug therapies from reaching their target in the TME while also providing cell proliferation, survival, migration, and differentiation [26]. Growth factors such as vascular endothelial growth factor (VEGF) and fibroblast growth factor (FGF), previously mentioned to be produced by endothelial cells and fibroblast cells, have been shown to sustain cell migration and proliferation while promoting angiogenesis [26]. Metabolic metabolites produced by the TME, such as increased lactate and pyruvate due to the Warburg effect, lead to increased production of adenosine triphosphate (ATP) while promoting tumor evasion [27]. Cytokines such as interleukins, interferons, tumor necrosis factors, and transforming growth factors function in both autocrine and paracrine mechanisms to increase tumor growth and drug resistance while facilitating recruitment, activation, and differentiation of other cells into the TME [28].

### 2.3. Mechanisms of TME Resistance to the Host Immune System

Host immune evasion and immunosuppression are critical to the survival and growth of malignant melanoma cells. Several studies have evaluated molecular areas of interest, including cytokines, prostaglandin E2 (PGE2), and myeloid-derived cells (MDSCs). Interferon-gamma (IFN-γ) has shown promise in immune cell defense by targeting tumor cells [29]. IFN-γ can be suppressed via cellular communication network factor 4 (CCN4), resulting in the inhibition of T-cell function [29]. However, IFN-γ has also been found to regulate the levels of nicotinamide phosphoribosyl transferase (NAMPT) via the upregulation of the interferon/signal transducer and activator of transcription 1 (IFN/STAT1) pathway, specifically a conserved enhancer on STAT [30]. In clinical mouse trials, increased IFN-γ activity induces NAMPT expression within the melanoma cells by enhancing the nicotinamide adenine dinucleotide (NAD+) salvage pathway, allowing greater energy and metabolic activity that enhances melanoma tumor growth29. BG34-200, a plant-derived carbohydrate molecule, binds to the CD11b integrin on monocytes to modulate cytokine levels, limiting the duration of pro-inflammatory cytokines such as IFN-γ activity [31].

PGE2 and 2-chloroadenosine (CADO) also play a role in melanoma progression via cytotoxic T-cell and lymphocytic immunosuppression, mediating the production of cyclic adenosine monophosphate (cAMP) and with resultant protein kinase A (PKA) activation when bound to their respective receptors [32]. An increase in the cAMP/PKA signaling pathway results in the suppression of downstream T-cell receptor (TCR) signaling, limiting T-cell maturation and promoting immunosuppression [32]. The melanocortin-1-receptor (MC1R) activates the guanine nucleotide-binding protein, alpha, stimulating protein kinase A (GNAS-PKA) signaling pathway to reduce the expression of chemokines and impair the infiltration of cytotoxic T-cells [8]. TILs are less effective against malignant melanoma cells in the absence of cytotoxic T-cells, with some studies suggesting a 50% reduction in effectiveness [32]. However, adhesion molecules such as intercellular adhesion molecule 1 (ICAM-1), vascular cell adhesion molecule 1 (VCAM-1), and selectins aid TILs in adhesion to epithelial cells and create a TME that favors the host cell in the fight against cancer-infiltrating cells [33]. ICAM-1 binds to LFA-1, whereas VCAM-1 binds to VLA-4 on effector T-cells. This binding results in strong surface cohesion of the T-cell to the endothelium of the tumor cell, allowing for rapid transmigration and a stronger immune response [33]. A defect in either ICAM-1 or VCAM-1 will result in decreased adhesion of the T-cell to the tumor epithelium, blunting the immunogenic response and resulting in resistance to therapy options.

Keratinocyte-derived thymic stromal lymphopoietin (TSLP) can also suppress cytotoxic T-cells to promote the growth and metastasis of melanoma by promoting GATA binding protein 3 (GATA3) expressing Tregs [34]. These Tregs express immune markers (such as PD-1) that suppress the activity of cytotoxic cell proliferation, facilitating tumor progression and metastasis [34]. However, uveal melanoma (UM) resulted in increased cytotoxic T-cell proliferation in comparison to skin cutaneous melanoma (SKCM). It was concluded that higher immune and stromal scores were associated with a decreased five-year survival and a poorer prognosis, and a high immune score correlated with a five-year survival rate of 40% and a higher cytotoxic T-cell count [35].

**Figure 3:**
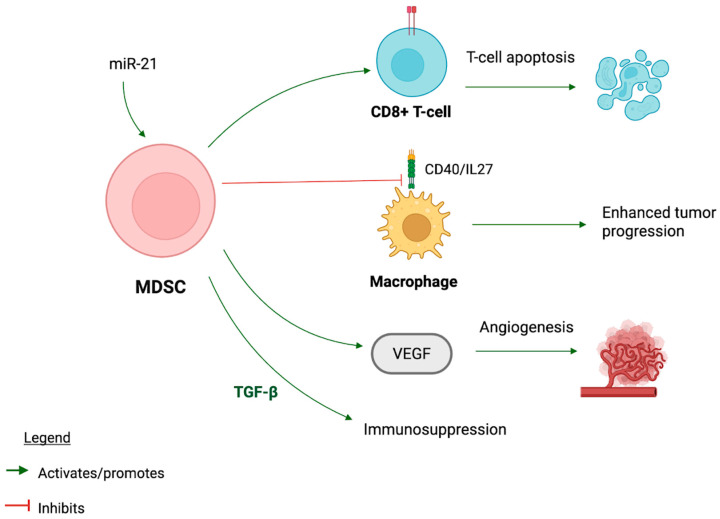
Myeloid-derived suppressor cell (MDSC) function and mechanism. This image was created in BioRender. Turner, L. (2025) https://app.biorender.com/illustrations/680f9ed03f4fb5dca3521d59?slideId=b4697751-4ea9-414c-a006-caba40c2f49b. This figure illustrates the role of MDSCs in promoting tumor progression within the TME. microRNA-21 (miR-21) promotes MDSC activation, which in turn induces CD8+ T-cell apoptosis, leading to immunosuppression. MDSCs inhibit macrophage activation via CD40 and interleukin-27 (IL-27) signaling, contributing to enhanced tumor progression. Additionally, MDSCs promote transforming growth factor-beta (TGF-β) release, which supports immunosuppression, and vascular endothelial growth factor (VEGF) release, promoting angiogenesis. Green arrows indicate activation or promotion, while red lines indicate inhibition.

Myeloid-derived suppressor cells (MDSCs) are a population of cells converted from myeloid cells that play a critical role in suppressing the host immune response within the TME. They are particularly active in chronic inflammatory states where they can inhibit the activation of CD8+ T cells and the overall immune response [36]. miR-155 and miR-21 have essential roles in MDSCs’ ability to secrete immunosuppressive cytokines and key signaling molecules [37]. Collectively, these signaling molecules have differing functions that induce either M1 or M2 macrophages, which greatly affect the tumorigenic response. miR-155 primarily functions to downregulate suppressor of cytokine signaling 1 (SOCS1), an immune checkpoint molecule that negatively regulates cytokine signaling. The downregulation of SOCS1 enhances T-cell proliferation and cytokine production, strengthening the immune response. miR-155 has also been found to upregulate DC populations, increase antigen presentation, and influence M1 macrophage proliferation to boost the pro-inflammatory response [18,37]. However, miR-21 targets and downregulates STAT in the IFN-γ signaling pathway, which is crucial for T-cell maturation and development. It also enhances the production of CAFs via the TGF-β signaling pathway and promotes an M2 macrophage shift, decreasing the effectiveness of the overall immune response to tumor cell invasion [18,37]. The impairment in T-cell function results in an immunosuppressive environment supportive of tumor progression, largely driven by enhanced CAFs and MDSCs [37]. MDSCs also increase VEGF and TGF-β in the TME, fostering a TME that promotes angiogenesis and expansion of melanoma cells via the suppression of cytotoxic T-cells [13]. MDSCs correlated with a 25% reduction in overall survival in patients with melanoma [4]. MDSCs also inhibit the CD40/IL-27 signaling in macrophages, increasing the risk of autoimmune conditions and tumor immunosuppression [38]. The neutralization of IL-27 via MDSCs’ inhibitory mechanism results in enhanced tumor progression, while blocking MDSCs results in IL-27 upregulation and delayed tumor progression [38]. Inhibition of phosphorylated protein eIF4E (phospho-elF4E) has shown inhibition of MDSCs and their immunosuppressive effects, a potential future immunotherapy target for the treatment of melanoma progression [39]. The various immunosuppressive mechanisms of MDSCs within the TME are summarized in Figure 3.

MDSCs derived from myeloid cells suppress the immune system in the TME by inducing apoptosis of CD8+ T cells, releasing immunosuppressive cytokine TGF-β, and releasing VEGF to promote tumor angiogenesis. 

Genetic factors can also heavily influence melanoma cell proliferation. Ras-related C3 botulinum toxin substrate 1(RAC-1)-mutant melanomas displayed a greater PD-L1 expression, resulting in T-cell inactivation and the absence of immune detection [40]. Insulin-like Growth Factor 2 messenger RNA (mRNA)-Binding Proteins (IGF2BP) are a broad category of proteins that regulate mRNA translation and are critical for the maintenance of mRNAs that enhance cell proliferation and survival. Downregulation of IGF2BP has been shown to make malignant melanoma cells more susceptible to immune cell destruction [41]. Additionally, tumor suppressor genes such as gene-encoding phosphatase and tensin homolog deleted on chromosome 10 (*PTEN*) can be mutated or suppressed, resulting in a poorer response to immunotherapy and, ultimately, an attenuated immune response; mRNA nanoparticle antitumor therapy is being studied to provide reactivation of this pathway [42]. KH-type splicing regulatory protein (KSRP) has also been found to be essential for melanoma cell proliferation in cells with and without acquired resistance to vemurafenib, a B-raf proto-oncogene (BRAF) kinase inhibitor [43]. Killin (KLLN), a DNA replication inhibitor regulated by the tumor suppressor gene p53, is a downstream effector of KSRP and is responsible for tumor suppression. KSRP suppresses KLLN anti-tumor activity via rapid mRNA decay, allowing for unregulated cellular proliferation to occur44. Decreased KLLN expression results in tumor cell advancement, including potential resistance to BRAF inhibitors. This demonstrates that targeting KSRP’s ability to suppress the tumor suppressor KLLN can have potential therapeutic effects in regulating melanoma growth. There are a variety of mechanisms by which melanoma evades the host immune system. Cellular immune interactions within the TME are largely responsible for the angiogenesis of melanoma cells and their progression [44]. Studies that continue to evaluate these interactions may provide insight into the future development of more effective treatments and may improve patient outcomes in melanoma and other forms of skin cancer. 

### 2.4. Resistance Mechanisms in the TME of Melanoma to ICIs—Decreased Host Immune Response 

Melanoma treatment using ICIs such as nivolumab and pembrolizumab (anti-PD-1) and ipilimumab (anti-CTLA-4 ) is very effective. As referenced above, these therapies target CTLA-4 and the PD-1/PD-L1 signaling axis to prevent co-inhibitory T cell signaling. However, widespread use of these therapies has resulted in resistance and melanomas that have adapted evasive mechanisms. Immunotherapy resistance is multifactorial, and TME, T cell infiltration, tumor profiles, and many other factors combine to determine the reaction patients may have to immune checkpoint therapy. 

#### 2.4.1. Metabolic Mechanisms of Resistance 

Activation and recruitment of CD8+ T cells are crucial to the effectiveness of ICIs: if T cells are prevented from infiltrating the TME, the therapies are ineffective and the tumor can persist or even metastasize. Tumor metabolic reprogramming, such as alterations in glucose, amino acids, and fatty acid metabolism, can play a critical role in immune evasion. For example, activation of β-catenin suppresses CD8+ T cell recruitment and contributes to primary resistance: by blocking cytotoxic T cell infiltration, the immune system is less effective in destroying melanoma cells [1]. Alterations in lactate, produced in anaerobic conditions, suppress CD8+ T cell activity and promote tumor resistance [27]. 

#### 2.4.2. Genetic and Epigenetic Mechanisms of Resistance

Resistance to ICIs in melanoma can arise from tumor-intrinsic genetic and epigenetic alterations that modify tumor antigen presentation and immune recognition. These changes create a TME where immune cells are either inhibited from infiltrating or rendered ineffective, leading to immune evasion and reduced efficacy of immunotherapies.

Increased PD-L1 expression by tumors is one prominent mechanism of resistance, especially in large tumors with activated IL-1α and the mitogen-activated protein kinase (MAPK) pathway [45]. PD-L1 overexpression allows melanoma cells to evade immune detection by binding to PD-1 on T cells, thus suppressing T cell activity and immune response. This mechanism underlies the failure of ICIs targeting the PD-1/PD-L1 axis. The β-catenin pathway, which can be activated in melanoma, further decreases ICI efficacy by suppressing CD8+ T cell recruitment into the TME [1]. β-catenin plays a critical role in immune resistance by altering the tumor’s ability to interact with immune cells. It blocks cytotoxic T cell infiltration and promotes an immunosuppressive environment that limits the effectiveness of ICIs. Furthermore, β-catenin in the TME is associated with increased activating transcription factor (ATF3) and decreased chemokine (C-C motif) ligand 4 (CCL4) production. The inverse correlation between β-catenin and CCL4 expression is partially explained by ATF3 suppressing CCL4 transcription, which prevents DC recruitment and contributes to a non-T-cell-inflamed phenotype [1]. Epigenetic modifications such as methylation of the TME can impact genes like beta-2-microglobulin (β2M) and Spi-1-proto-oncogene (SPI1), which control CD1D expression that is crucial for tumor antigen presentation and immune recognition [46]. By silencing these genes, proper MHC-I expression is prevented and antigen presentation is impaired, which results in a decreased ability for immune cells, particularly CD8+ T cells, to recognize and attack melanoma cells. N6-methyladenosine RNA methylation works similarly and decreases ICI efficacy by suppressing immune cell recruitment [47]. 

Enhancer of zeste homolog 2 (EZH2), a histone modifier, further contributes to resistance by suppressing MHC-II presentation, which is vital for T cell recognition and activation in the TME [48]. Through its effects on histone modifications, EZH2 regulates gene expression that directly affects immune cell recognition and alters the overall immunogenicity of melanoma cells, allowing the tumor to evade immune surveillance. Inactivation of F-box and WD repeat domain-containing 7 (FBXW7) reduces the expression of dsRNA sensors like MDA5 and RIG-I, which are involved in recruiting IFN and inducing MHC-I expression [49]. This impairs the immune response by reducing the production of interferons that are essential for immune cell activation and antigen presentation. Additionally, this alteration within the TME disrupts the immune response, contributing to immune evasion and resistance to ICIs. The microphthalmia-associated transcription factor (MITF) regulates antigen presentation and the expression of co-inhibitory receptors in melanoma cells, creating an immune environment that limits the infiltration of immune cells like TILs [50]. By controlling inflammatory cytokine production and modifying the tumor’s immune profile, MITF contributes to a TME that is less responsive to immunotherapy, reducing the effectiveness of ICIs in melanoma treatment.

Another key transcription factor, transcription factor 4 (TCF4), promotes a mesenchymal-like state in melanoma cells that reduces susceptibility to T cell infiltration, downregulates antigen presentation, and impairs interferon signaling, all of which are critical for ICI resistance [51]. By altering the tumor’s phenotype, TCF4 enables melanoma cells to survive and proliferate in an immune-suppressive environment, making them more resistant to immune checkpoint blockade. Furthermore, zinc finger E-box binding homeobox 1 (ZEB1), a transcription factor, further diminishes ICI efficacy by suppressing the excretion of CXCL10, a chemokine essential for CD8+ T cell recruitment [52]. ZEB1 impairs the recruitment of immune cells into the TME by downregulating CXCL10 production, which is necessary for T cells to enter the tumor and attack melanoma cells [52]. This creates a non-T-cell-inflamed phenotype, often associated with poor responses to immunotherapy. These genetic and epigenetic alterations collectively reshape the TME to favor tumor progression and immune evasion, preventing the successful application of ICIs. By altering antigen presentation, immune cell recruitment, and the tumor’s immunogenicity, these mechanisms facilitate melanoma’s ability to resist immune checkpoint therapy and thrive in an otherwise immune-competent host. A summary of genetic and epigenetic pathways that contribute to immunotherapy resistance can be found below in Table 1.

#### 2.4.3. Immune Cell Recruitment and Immune Suppression

In melanoma, immune evasion is driven by multiple molecules that suppress immune cell recruitment and activity as shown in Table 2. Activin-A impedes T cell recruitment by decreasing the release of CXCL9/10 chemokines, essential for CD8+ T cell infiltration into the TME [53]. C-C chemokine receptor type 2 (CCR2) macrophages infiltrate tumors, creating an immunosuppressive environment that supports melanoma survival by releasing pro-tumor cytokines [54]. The presence of MDSCs within the TME is another critical factor in immune suppression, as these cells inhibit T cell activity through the secretion of TGF-β, while Tregs suppress CD8+ T cells through PD-1 expression [55,56]. Additionally, interleukin-1-alpha (IL-1α) increases PD-L1 expression on melanoma cells, further aiding in immune evasion by preventing immune detection and activity [45].

Further enhancing immune suppression, PD-1 promotes the recruitment of mast cells, which release histamine and cytokines, further inhibiting the immune response and fostering an immunosuppressive environment within the TME [57]. Additionally, the programmed cell death ligand 1/NOD-, LRR-, and pyrin domain containing protein 3 (PD-L1/NLRP3) inflammasome pathway recruits polymorphonuclear myeloid-derived suppressor cells (PMN-MDSCs) that mediate apoptosis in TILs (tumor-infiltrating lymphocytes), further preventing T cell activity [58]. The T cell immunoreceptor with Ig and ITIM domains (TIGIT)/CD55 axis contributes to immune suppression by suppressing T cell recruitment and activation, thereby reducing the immune system’s ability to target melanoma cells effectively [59]. Additionally, thymus leukemia stromal protein (TSLP) suppresses CD8+ T cell activity, further enhancing the immunosuppressive environment in the TME and aiding ICI resistance [34].

#### 2.4.4. Tumor Cell Survival and Proliferation

Tumor cells in melanoma also enhance their survival through intrinsic mechanisms that reduce immune surveillance. Ambral protein loss facilitates autophagy, apoptosis, and cell proliferation, contributing to metastatic spread and immune evasion [60]. AXL receptor tyrosine kinase (AXL kinase) plays a key role by upregulating PD-L1 expression on tumor cells, inhibiting NK cell activation, and supporting tumor survival [61]. Baculoviral IAP repeat containing 2 (BIRC2) further contributes to resistance by suppressing CXCL9 excretion, which limits CD8+ T cell recruitment [62]. Similarly, nerve growth factor receptor (NGFR) induces brain-derived neurotrophic factor (BDNF), which allows melanoma cells to evade T cell killing mechanisms, supporting tumor progression [63]. Rho-associated protein kinase-Myosin II pathway (ROCK-Myosin-II pathway) enhances tumor cell survival by preventing DNA damage and reducing reactive oxygen species (ROS) [64]. In melanoma, genetic alterations also support tumor progression and survival. ZEB1 transcription factor contributes to resistance by suppressing the excretion of the CXCL10 chemokine, inhibiting CD8+ T cell recruitment, and favoring immune evasion [52]. Tumor cell survival and proliferation mechanisms contributing to decreased therapy response in melanoma have been summarized in Table 3.

#### 2.4.5. Immune Escape Through Reprogramming

Melanoma cells modify the TME to evade immune detection and promote tumor progression. The creation of a fibrotic stroma ECM shield around melanoma cells facilitates MMP9-dependent PD-L1 cleavage, downregulating MHC-I expression and helping the tumor evade immune recognition [65,66]. Senescent cells contribute by recruiting MDSCs, further inhibiting immune cell function, and enabling tumor resistance to ICIs [67]. The Fas-L pathway induces TIL apoptosis, which eliminates critical immune cells and allows melanoma cells to escape immune surveillance [68]. The Glioma-Associated Oncogene family zinc finger 2 (GLI2) pathway recruits PMN-MDSCs and impairs the function of DCs, CD8+ T cells, and NK cells, resulting in a suppressed immune response and aiding in resistance to ICIs [69]. Hepatocyte Growth Factor-Regulated Tyrosine Kinase Substrate (HRS) phosphorylation impedes T cell recruitment by enhancing PD-L1 interactions and modifying the TME [70]. IL4I1 reduces CD8+ T cell infiltration by depleting essential amino acids and generating toxic metabolites like H2O2 and indole, further impairing immune responses [71]. The MAPK pathway increases PD-L1 expression on melanoma cells, reducing T cell function and promoting immune evasion [72]. Peroxynitrite alters major histocompatibility complex class 1 (MHC-I) antigen presentation, preventing immune cells from recognizing melanoma cells and contributing to immune resistance [73]. Additionally, prostaglandin E2/2-chloroadenosine (PGE2/CADO) increases cAMP and protein kinase A (PKA), which reduces the effectiveness of TILs by impairing their ability to infiltrate and respond to melanoma cells [32].

Resistance to ICIs in melanoma is driven by a combination of metabolic, genetic, epigenetic, and cellular factors that create a dynamic and evolving TME. The complex interactions between altered metabolic pathways, genetic modifications, epigenetic reprogramming, and immune cell dynamics complicate the therapeutic response. Understanding these mechanisms can help inform the development of more effective therapies, including combination approaches that target these specific resistance pathways. By targeting metabolic reprogramming, genetic alterations, and immune suppression within the TME, future melanoma therapies may overcome the current limitations of ICI treatment and provide more personalized and effective treatment options for melanoma patients. Immune escape reprogramming mechanisms contributing to decreased therapy response in melanoma have been summarized in Table 4.

## 3. Overcoming Immunotherapy Resistance and Increasing Host Response

As discussed earlier, resistance to immunotherapy in melanoma remains a complex challenge. However, ongoing research has identified many key pathways and interventions that can help overcome this resistance and improve the response to ICIs, such as anti-PD-1 and anti-CTLA-4. These interventions can be grouped into four primary categories: Immune Cell Modulation, Combination Therapies, Tumor Metabolism Targeting, and Signaling Pathway Inhibition. By addressing these areas, novel therapies can enhance T cell recruitment, increase immune activity, and target the mechanisms that allow melanoma cells to evade immune surveillance.

### 3.1. Tumor Metabolism Targeting

Metabolic reprogramming within the TME significantly contributes to immunotherapy resistance. Lactate produced by lactate dehydrogenase (LDH) creates an acidic, immunosuppressive environment, impairing T cell function and promoting tumor evasion. LDH inhibition reduces lactate production, which restores T-cell function and enhances immune responses [75]. Similarly, ALK-04, which inhibits α-ketoglutarate-dependent dioxygenase AlkB homolog 5 (ALKBH5) and monocarboxylate transporter 4 (MCT4), decreases lactate secretion, reversing immunosuppressive effects and improving immune activation in the TME [76]. Myeloperoxidase (MPO) inhibition is another promising strategy for improving ICI efficacy. MPO generates ROS in the TME, contributing to immune suppression. By inhibiting MPO, ROS production is reduced, leading to enhanced immune cell infiltration and improved tumor control [77]. Shifting tumor metabolism toward glycolysis by inhibiting tricarboxylic acid (TCA) cycle enzymes enhances the efficacy of anti-PD-L1 therapy, sensitizing tumors to immune-mediated killing and improving overall treatment response [78]. SGN1, a genetically modified Salmonella typhimurium, induces methionine deprivation, improving immune cell infiltration within the TME. Its effects are further potentiated when combined with PD-L1 inhibitors, making it a promising adjunct to immunotherapy for increasing therapeutic response [79].

Tumor metabolism pathway targeting strategies are summarized below in Table 5.

### 3.2. Interventions Involving T Cell Activity or Infiltration 

To overcome resistance to ICIs and enhance host immune responses in melanoma, several therapeutic strategies aim to improve T cell activity and infiltration within the TME. These interventions can be categorized into those that increase T cell activation and proliferation, improve immune cell recruitment, and reduce immunosuppressive factors in the TME.

One intervention involves A0317859, which inhibits p21-activated kinase 4 (PAK4). This leads to enhanced T cell and CD103+ DC infiltration into tumors, improved blood vessel function, and increased CCL21 levels. This combination has been shown to improve ICI efficacy and prolong survival [80]. Inhibition of the protein A20 has also been linked to increased CD8+ T cell activity, which enhances the effects of PD-1 therapy in therapy-resistant tumors [81]. IL-2, a well-known cytokine, has been used in combination with anti-PD-1 therapy to significantly expand CD8+ T cell numbers and function. This combination, when administered via gene electrotransfer (GET), improves MHC Class I presentation on tumor cells, leading to enhanced antigen presentation and increased production of IFN-γ and granzyme, thereby enhancing the immune response [82,83]. Interleukin-32 gamma (IL-32γ) activates intratumoral DCs and macrophages, inducing chemokines such as CCL5 and CCL4, which recruit and activate CD8+ T cells. Elevated levels of IL-32γ have been associated with better responses to anti-PD-1 therapy and improved survival outcomes [84].

In addition to enhancing T cell activation, improving immune cell recruitment to the tumor site is a critical step. CCL4, a chemokine, plays a significant role in promoting T cell recruitment into the tumor, thereby enhancing the overall immune response [1]. CXCR3 and its ligand CXCL9 are essential for CD8+ T cell activation and proliferation within the TME [85]. Enhancing CXCL9 expression has been shown to improve melanoma tumor responsiveness to ICIs, demonstrating the importance of these molecules in overcoming resistance [86]. Cross-presenting dendritic cells (cDC1s), a subset of DCs, are important for T and NK cell priming. Increased presence of cDC1s in the TME is linked to improved T cell activation, contributing to stronger anti-tumor immunity. These DCs, in combination with dendritic mesoporous organosilica nanoparticles carrying double-stranded DNA (dsDNA@DMONs), increase type-I interferon production and DC maturation, which further enhances T cell activation and improves ICI response [87]. Furthermore, engineered exosome-like nanovesicles modified with the fibroblast activation protein-α gene (eNVs-FAP) have been shown to enhance DC maturation, reduce immunosuppressive cells, and increase cytotoxic T lymphocytes (CTLs), which can improve melanoma immunotherapy outcomes [88].

Targeting immunosuppressive factors within the TME is equally important for enhancing immune responses. MDSC inhibition targets MDSCs, which deplete amino acids, produce ROS, and release inhibitory molecules like TGF-β and IL-10, all of which suppress T cell function. Inhibiting MDSCs, particularly in combination with ICIs, has been shown to improve immune responses in melanoma [36]. Triggering receptor expressed on myeloid cells (TREM1) inhibition reduces MDSC-mediated suppression, increases CD8+ T cell activity, promotes neutrophil infiltration, and decreases IL-18 signaling, thus improving T cell function [89]. CD96 inhibition has been shown to increase the CD8+ T cell/Treg ratio and reduce myeloid cell-mediated immune suppression, further enhancing T cell activity within the TME [90]. Sphingosine kinase 1 (SK1) inhibitors reduce immunosuppressive cytokines such as TGF-β and IL-10, while enhancing CD8+ T cell function and the tumor’s response to ICI [91]. Ferredoxin-1 (FDX1) inhibition has been associated with increased CD8+ T cell infiltration and a reduction in immunosuppressive cell populations in the TME, thereby enhancing anti-tumor immunity [92]. Renalase (RNLS) inhibitors inflame the TME, reduce T-reg populations, and enhance immune cell infiltration, leading to greater tumor shrinkage when combined with anti-PD-1 therapy [93].

In addition to modulating immune cells, targeting tumor resistance pathways has shown potential in improving immune responses. CDK6 depletion inhibits tumor growth and increases T and NK cell activity by reducing the suppression of T cell receptor signaling, which is crucial for immune recognition of melanoma cells [94]. β-catenin inhibition increases T cell recruitment by enhancing CCL4 production, preventing immune evasion, and improving responses to ICIs [1]. Uncoupling protein 2 (UCP2) reprograms the TME from immunosuppressive to immunostimulatory by increasing CXCL10 production, attracting T cells to the tumor, and normalizing tumor vasculature, which further improves T cell infiltration [95]. 

These interventions (targeting T cell activation, immune cell recruitment, and immunosuppressive factors) are critical in overcoming melanoma resistance to ICIs. By altering the TME to support immune activation and counteracting resistance mechanisms, these therapies may improve melanoma treatment outcomes. Interventions targeting T cell activity or immune cell infiltration are summarized below in Table 6.

### 3.3. Interventions Involving Macrophages or Other Molecules and Pathways

Overcoming immunotherapy resistance in melanoma requires targeting several molecules and pathways that regulate immune cell activity and tumor progression. Among these, macrophages and other tumor-associated molecules play pivotal roles in modulating the TME and influencing the effectiveness of ICIs. One approach involves the inhibition of the R-spondin-Leucine-rich repeat containing G-protein coupled receptor 4 (R-spondin/LGR4) signaling, which is crucial for pro-tumor M2 macrophage polarization. Blocking this pathway prevents macrophage polarization towards an immunosuppressive M2 phenotype and instead promotes an anti-tumor M1 phenotype, which increases CD8+ T cell activity and enhances the response to anti-PD-1 therapy [96].

Similarly, histone deacetylase (HDAC) inhibition via low-dose trichostatin-A (TSA) can reprogram macrophages from a pro-tumor M2 phenotype to a more immune-activating M1 phenotype [97]. This reprogramming of TAMs also reduces MDSCs, which play a major role in suppressing the immune response. This process improves the efficacy of anti-PD-1 therapy [97]. In addition, the inhibition of STAT3 has been shown to decrease the expression of T cell immunoglobulin and mucin domain-containing protein 3 (TIM3) on Treg cells, which is linked to reduced immunosuppressive cytokine production, particularly IL-10 and TGF-β. This intervention helps to reduce the immune suppressive effects in the TME and enhances the anti-tumor immune response when combined with ICIs [98].

Another approach to overcoming immunotherapy resistance is through phosphatidylinositol 3-kinase catalytic subunit type 3 (PIK3C3)/vacuolar protein-sorting 34 (VPS34) inhibitors. These inhibitors work by switching “cold” tumors, which are less responsive to immunotherapy, into inflamed tumors. This transformation occurs through the increased production of pro-inflammatory cytokines such as CCL5 and CXCL10. These cytokines help recruit and activate immune cells like NK and CD8+ T cells, enhancing the host’s immune response to melanoma [99]. However, the effectiveness of this intervention depends on the presence of these immune cells, as depletion of NK and CD8+ T cells significantly reduces the efficacy of PIK3C3/VPS34 inhibitors [99]. The tumor suppressor gene p53 is also a potential target for overcoming resistance: p53 increases activation of IL-15 and MHC-II, allowing for improved antigen presentation [100]. 

Further, the role of MAFs in the TME is critical to the progression of melanoma and resistance to ICIs. MAFs are known to secrete immunosuppressive cytokines, such as IL-10, and promote tumor growth by inducing neutrophil extracellular traps (NETs), which contribute to T cell exhaustion [101]. However, subsets of MAFs, such as CD105-negative CAFs and CXCL13-positive MAFs, inhibit tumor growth and reduce T cell exhaustion. These specific subsets of fibroblasts counteract the immunosuppressive effects typically associated with the TME [101]. This modulation of MAFs and other molecules in the TME provides a strategy for enhancing the efficacy of ICIs and overcoming resistance in melanoma patients.

In summary, combining these interventions targeting macrophage polarization, signaling pathways, and fibroblast subsets offers a complex approach to overcoming immunotherapy resistance. These strategies are essential for improving melanoma treatment responses and advancing personalized therapies. Interventions involving macrophages or other molecules have been summarized below in Table 7.

### 3.4. Melanoma Immunotherapy and Resistance Overview

Immunomodulatory checkpoint inhibitors discussed in this review include monoclonal antibodies against CTLA-4, PD-1, and programmed cell death ligand 1(PD-L1). These inhibitors block immunosuppressive receptors on activated T cells, which are involved in tumor surveillance at the interface between tumor cells, the local tumor environment, and the host immune system [5]. PD-1 is a transmembrane protein expressed on activated T cells, B cells, and other immune mediators, and binds to PD-L1 or PD-L2 on antigen-presenting cells to inhibit T cell signaling and promote tumor evasion in cancer [102]. However, cancer cells overexpress PD-L1 and can induce “T-cell exhaustion”, or dysregulation by chronically stimulating T cells over time and causing impaired glycolysis and mitochondrial processes [102]. The PD-1 and PD-L1 interaction has also been shown to promote tumor progression in the TME by promoting Treg development, and is thought to promote immunosuppressive M2 macrophage polarization [103]. By inhibiting PD-1 or PD-L1, T cell activity and cytotoxicity are restored, and tumor cells are more sensitive to immune checkpoint therapy. For example, nivolumab and pembrolizumab (PD-1 checkpoint inhibitors approved for use in melanoma) have been shown to decrease Tregs and improve anti-tumor response [104]. CTLA-4 is a checkpoint protein found on T cells and binds the B7 protein on antigen-presenting cells. CTLA-4:B7 binding results in decreased T cell activation by preventing the normally present costimulatory signal of CD28:B7 and limiting interleukin-2 (IL-2) proliferation [105]. Ipilimumab, an anti-CTLA-4 monoclonal antibody approved for use in melanoma, has been shown to prolong survival. Overall, nivolumab (anti-PD-1), pembrolizumab (anti-PD-1), and ipilimumab (anti-CTLA-4) have shown sustained tumor regression, decreased recurrence rates, and prolonged overall survival in melanoma [106,107]. However, subsequent widespread use and longitudinal data surrounding ICIs have revealed observations of a lack of efficacy initially and over time via both primary and secondary resistance mechanisms [5]. Primary resistance indicates that a patient did not respond to initial therapy, while others develop secondary or acquired resistance via recurrences [108]. For example, the absence of T-cell infiltration in melanoma has been correlated with primary resistance to ICIs, such as PD-1 and PD-L1 blockers [2]. 

Patel et al. have described overarching themes of resistance mechanisms and suggest that melanoma responds to targeted therapies and ICIs in 3 phases: early response, minimal residual disease, and disease progression [109]. Minimal residual disease allows for the development of acquired resistance that gives rise to new tumors that may be refractory to the original treatment [109]. Melanoma is known for its high mutational burden, and tumor progression or recurrence, despite immunotherapy, continues to be a highly complex and prevalent issue [110]. There are many proposed mechanisms of melanoma immunotherapy, both broad and specific. Resistance concepts on a larger scale include intratumoral heterogeneity, cellular plasticity, phenotype switching, depletion of immune cell metabolites, and upregulation of suppressive T cell populations that allow for tumor adaptation and survival [71,111,112]. 

One of the most prevalent topics discussed is the concept of “hot” versus “cold” tumors, referring to high and low infiltration of immune cells within the TME [113] “Cold”, or non-inflamed tumors, typically show minimal response to therapy, while “hot” or inflamed tumors, show better response due to a higher tumor mutational burden (TMB), increased neoantigens, and increased expression of PD-L1 [113]. 

A few of the most frequently reported molecular resistance players include pathways involving metabolic, transcriptomic, epigenetic, cytokine, growth factor, MDSC, and RNA signaling pathways [8,41,89,114,115,116].

## 4. Materials and Methods

This narrative literature review evaluates the resistance mechanisms behind the TME in melanoma, which allows cancer to evade ICIs used in the treatment of cutaneous malignancies. This review was not conducted in accordance with PRISMA guidelines, and no formal quality assessment of included studies was performed. 

A systematic approach was used to gather as much knowledge and research on the topic while simultaneously enhancing the internal and external validity of this study. Keywords and phrases that included [“melanoma AND immune checkpoint inhibitor resistance”] were put into the PubMed database search criteria to select the relevant literature for this review. Filters were applied to restrict the language to articles published in English, and articles published within the last 10 years at the time of our literature search (July 2024). No other filters were applied. PubMed was selected as the primary database for this review to limit the scope of the review and reduce the number of articles that required screening. Given our constraints in terms of resources and team size, we chose to use PubMed to effectively streamline the screening process. While we recognize that restricting this narrative review to a single database can limit the comprehensiveness of this paper, we decided on this approach to best maintain a feasible dataset. This limitation has the potential to impact the breadth of our review, and we suggest that future research incorporate additional databases for an increased range of applicable studies. 

The relevant literature identified in PubMed was uploaded onto Covidence, a software used to conduct title/abstract screening, full-text screening, data abstraction, and quality assessment among literature reviewers. Covidence was used as a tool to help organize and delineate relevant studies for this narrative review. 

A total of 1052 studies were extracted from the PubMed database. These studies then underwent title/abstract screening amongst two literature reviewers, with a third literature reviewer resolving conflicts. Approximately 373 studies passed the title/abstract screening. Two additional reviewers completed the full-text screening, and after a full-text review, 101 studies were identified that fit the inclusion/exclusion criteria. 

The following criteria were included for the study: review articles, empirical studies, and meta-analysis papers published in peer-reviewed journals, papers written in English that were published between 2014–2024, and study topics that focused on melanoma TME resistance mechanisms to ICIs, current concurrent ICI therapy used as either monotherapy or in concurrence with another immunotherapy and aspects of the TME that can be a future target for ICIs. Exclusion criteria included studies without full-text accessibility, non-peer-reviewed articles, and article topics with a focus on resistance mechanisms and immunotherapies not specific to melanoma, as well as mono-immunotherapies that were not ICIs.

## 5. Conclusions

With the robust production of literature surrounding melanoma TME and immunotherapy resistance in recent years, many have suggested possible outlets for combating this resistance, as evidenced by this review. Existing research and reviews have suggested the development of targeted therapies as well as individualized treatments to definitively manage melanoma on a case-by-case basis. This requires categorizing patients by their tumor characteristics, such as T cell infiltration, and intrinsic signaling pathways, to know whether a treatment response is predictable. Furthermore, there is a need to identify validated biomarkers that can accurately predict melanoma response before subjecting patients to therapies with both adverse effects and little efficacious potential [2,7]. Predictive biomarkers and specific models are the future of individualized melanoma management and show great promise in their approach to targeted therapy production [10]. Tumor profiling can be utilized to help predict the efficacy of ICIs, and specific biomarkers predicting therapy responses are instrumental in moving towards personalized and more efficacious medicine. As more melanoma resistance emerges, alternative and combinatorial therapy aligned with knowledge of existing resistance mechanisms will be needed. 

Monotherapies and adjuvant treatments in combination with immunotherapies have been suggested in the forms of chemotherapy, radiotherapy, metabolic modulators, photodynamic therapy (PDT), cytokines, oncolytic viruses, and other novel targeted therapies to revert intrinsic resistance and resensitize the tumor environment to immunotherapy [117,118,119]. There are also emerging studies that correlate a relationship between the gut microbiota and melanoma cell progression [120]. Ultimately, numerous pathways are involved, with discoveries yet to be made regarding extensive resistance mechanisms to ICI therapies for melanoma. 

## Figures and Tables

**Table 1:** Genetic and Epigenetic Molecules and Pathways that Decrease Immune Response in Melanoma.

Molecule/Pathway	Mechanism
β-catenin pathway	Decreases ICI efficacy by suppressing CD8+ T cell recruitment [1].
Epigenetic modifications	Methylation of TME impacts β2M and SPI1, which control CD1D expression and affect tumor antigen presentation [46].
EZH2 pathway	Suppresses MHC-II presentation [48].
FBXW7 inactivation	Decreases expression of dsRNA sensors (MDA5 and RIG-I), which recruit IFN and cause MHC-I expression, altering the TME [49].
MITF transcription factor	Changes antigen presentation, controls expression of co-inhibitory receptors, and inflammatory secretome production to reduce infiltration of TME [50].
N6-methyladenosine RNA methylation	Decreases ICI efficacy by suppressing immune cell recruitment [47].
TCF4 transcription factor	Promotes a mesenchymal-like state in the tumor, which causes low susceptibility to T cell infiltration, downregulates antigen presentation, and interferon signaling, causing resistance [51].
ZEB1 transcription factor	Decreases ICI efficacy by suppressing the excretion of the CXCL10 chemokine that recruits CD8+ T cells, leading to suppressed T cell recruitment [52].

**Table 2:** Immune Cell Recruitment and Immune Suppression Mechanisms Contributing to Decreased Therapy Response in Melanoma.

Molecule/Pathway	Mechanism
Activin-A	Prevents T cell recruitment by decreasing CXCL9/10 chemokine release [53].
CC2R macrophages	Infiltrate tumors and help support drug resistance [54].
IL-1a	Increases PD-L1 expression on tumors [45].
MDSCs	Correlated with the MAPK pathway and increased PD-L1 expression. MDSCs also work to decrease the host immune response via increased levels of IL-6, VEGF, and TGF-β to induce T-cell apoptosis. They also neutralize the IL-27/CD40 signaling pathway [38,55].
PD-1	Recruit mast cells, which release histamine and cytokines to alter the TME [57].
PD-L1/NLRP3 inflammasome pathway	Recruits PMN-MDSCs with high Fas/FasL levels, which mediate apoptosis [58].
TIGIT/CD55 axis	Decreases ICI efficacy by suppressing T cell recruitment [59].
TLSP	Suppress CD8+ T-cells [34].
Tregs	Tregs create an immunosuppressed TME by reducing CD8 T cell granzyme B and increasing PD-1 [56].

**Table 3:** Tumor Cell Survival and Proliferation Mechanisms Contributing to Decreased Therapy Response in Melanoma.

Molecule/Pathway	Mechanism
Ambral protein loss	Loss of TME maintenance facilitates autophagy, apoptosis, cell proliferation, and invasion. This promotes metastasis and accelerated growth [60].
AXL kinase	Creates an immunosuppressed environment by upregulating PD-L1 on tumors and downregulating NK cells [61].
BIRC2	Decreases ICI efficacy by suppressing the excretion of CXCL9 chemokine that recruits CD8+ T cells [62].
NGFR	Induces BDNF, which allows them to evade T cell killing mechanisms [63].
ROCK-Myosin-II pathway	Diminishes reactive oxygen species and prevents DNA damage in cancerous cells [64].
ZEB1 transcription factor	Decreases ICI efficacy by suppressing the excretion of the CXCL10 chemokine that recruits CD8+ T cells, leading to suppressed T cell recruitment5 [3].

**Table 4:** Immune Escape Reprogramming Mechanisms Contributing to Decreased Therapy Response in Melanoma.

Molecule/Pathway	Mechanism
Fas-L	High levels of the Fas/Fas-ligand mediate apoptosis of TILs [68].
Fibrotic Stroma	The tumor creates a fibrotic stroma ECM shield in response to therapy and protects itself through MMP9-dependent PD-L1 cleavage and downregulation of MHC-I expression [65,74].
GLI2 Pathway	Decreases host immune response by recruiting PMN-MDSCs and impairing the function of DCs, CD8+T cells, and NK cells [69].
HRS phosphorylation	Decreases ICI efficacy by suppressing T cell recruitment, enhancing the effect via interaction with PD-L1 [70].
IFN-γ/NAMPT	Decreases host immune response by upregulating the IFN/STAT1 pathway, which increases NAMPT levels and allows for greater tumor growth [30].
IL4I1	Reduces CD8+ T cell infiltration and creates an immunosuppressive environment by depleting amino acids essential for T cells, producing toxic metabolites H_2_O_2_ and indole, and activating the aryl hydrocarbon receptor [71].
MAPK Pathway	Increases PD-L1 expression on tumors [72].
Peroxynitrite	Presence causes alteration of MHC-I antigen presentation on tumor cells [73].
PGE2/CADO	Increase cAMP and PKA, decreasing the effectiveness of TILs [32].

**Table 5:** Tumor Metabolism Targeting Pathways in Melanoma.

Molecule/Pathway	Mechanism
ALK-04	Inhibits ALKBH5 and MCT4, reducing lactate levels [76].
Lactate dehydrogenase (LDH) inhibition	Reduces lactate production, restores T cell function [75].
Myeloperoxidase inhibition	Prevents the production of ROS [77].
SGN1	Induces methionine deprivation, improves immune cell infiltration [79].
TCA cycle enzyme inhibition	Shifts metabolic preference to glycolysis [78].

**Table 6:** Interventions Involving T Cell Activity or Infiltration to Overcome Immunotherapy Resistance.

Molecule/Pathway	Mechanism
A0317859	Inhibits PAK4, improves blood vessel functionality, and increases immune cell tumor infiltration by increasing CCL21 levels [80].
A20 inhibition	Increases CD8+ T cell activity [81].
CCL4	Increased T cell recruitment and infiltration [1].
cDC1s	Increases NK and T cell priming [86].
CDK6 depletion	Inhibits tumor growth, increases T and NK cell activity [94].
CD96 inhibition	Increases the CD8+ T cell/Treg ratio, decreases myeloid cell-mediated immune suppression [90].
CXCR3 and CXCL9	Essential for the activation and proliferation of CD8+ T cells within the TME [85,86].
dsDNA@DMONs	Upregulate IFN-Is, increase maturation of DCs, and increase T cell activation [87].
ENVs-FAP	Increase activation and maturation of DCs, and increase CTLs [88].
FDX1 inhibition	Increases CD8+ T cells, decreases immunosuppressive cells, involved in lactate metabolism [92].
IL-2	Increases T-cell activation and proliferation, improves antigen presentation, and increases IFN-γ and granzyme production [82,83].
IL-32γ	Activates intratumoral DCs, increases CD8+ T cells [84].
MDSC inhibition	Restores amino acids, prevents production of ROS, TGF-B, and IL-10 [36].
RNLS inhibition	Reduce T-regs, increase immune cell infiltration [93].
SK1 inhibitors	Reduces immunosuppressive cytokines, improves CD8+ T cell function [91].
TREM1 inhibition	Increases CD8+ T cell activity, reduces MDSC immunosuppression [89].
UCP2	Increases CXCL10 production to attract T cells, normalizes tumor vasculature [95].

**Table 7:** Interventions Involving Macrophages or Other Molecules and Pathways to Overcome Immunotherapy Resistance.

Molecule/Pathway	Mechanism
CD105 and CXCL13+ MAFs	Reduce T cell exhaustion, prevent activation of MDSCs [101].
PIK3C3/VPS34 inhibitors	Increased production of pro-inflammatory cytokines CCL5 and CXCL10 [99].
p53	Tumor suppressor gene that increases activation of IL-15 and MHC Class II [100].
R-spondin/LGR4 inhibitors	Prevents pro-tumor M2 macrophage polarization, increases CD8+ T cells [96].
STAT3 inhibition	Decreases TIM3 expression, reduces immunosuppressive cytokine production (IL-10 and TGF-β) [98].
TSA-induced HDAC inhibition	Reprograms pro-tumor M2 phenotype to anti-tumor M1 phenotype in macrophages, reduces MDSCs [97].

## Data Availability

No new data were created or analyzed in this study. Data sharing is not applicable to this article.

## Abbreviations

ALKBH5	α-ketoglutarate-dependent dioxygenase AlkB homolog 5
ATF3	activating transcription factor
ATP	adenosine triphosphate
AXL	AXL receptor tyrosine kinase
BDNF	brain-derived neurotrophic factor
BIRC2	baculoviral IAP repeat containing 2
BRAF	B-raf proto-oncogene
β2M	beta-2-microglobulin
CAF	cancer-associated fibroblast
cAMP	cyclic adenosine monophosphate
cDC1	Conventional type 1 dendritic cell
CADO	2-chloroadenosine
CCL	chemokine (C-C motif) ligand
CCN4	cellular communication network factor 4
CCR2	C-C chemokine receptor type 2
CTLA-4	cytotoxic T-lymphocyte-associated protein 4
CTL	cytotoxic T-lymphocyte
CXCL1	chemokine (C-X-C motif) ligand 1
DC	dendritic cell
dsDNA@DMON	dendritic mesoporous organosilica nanoparticles carrying double-stranded DNA
ECM	extracellular matrix
EM-DR	environment-mediated drug resistance
eNVs-FAP	engineered exosome-like nanovesicles modified with the fibroblast activation protein-α gene
EZH2	enhancer of zeste homolog 2
FBXW7	F-box and WD repeat domain-containing 7
FDX1	erredoxin-1
FGF	fibroblast growth factor
FOXP3	forkhead box P3
GATA3	GATA binding protein 3
GET	gene electrotransfer
GLI2	Glioma-Associated Oncogene Family Zinc Finger 2
GNAS-PKA	guanine nucleotide-binding protein, alpha stimulating protein kinase A
HDAC	histone deacetylase
HRS	Hepatocyte Growth Factor-Regulated Tyrosine Kinase Substrate
ICI	immune checkpoint inhibitor
ICAM-1	intercellular adhesion molecule 1
IFN	interferon
IFN-γ	interferon gamma
IGF2BP	insulin-like growth factor 2 mRNA-binding protein
IL-32γ	interleukin-32 gamma
IL4I1	interleukin-4 induced-1
KLLN	killin
KSRP	KH-type splicing regulatory protein
LDH	lactate dehydrogenase
LIF	leukemia inhibitory factor
MAF	melanoma-associated fibroblast
MAPK	mitogen-activated protein kinase
MCT4	monocarboxylate transporter 4
MC1R	melanocortin-1-receptor
MDSC	myeloid-derived suppressor cell
MHC	major histocompatibility complex
MITF	microphthalmia-associated transcription factor
MiR	micro RNA
MMP	matrix metalloproteinase
mRNA	messenger ribonucleic acid
NAD+	nicotinamide adenine dinucleotide
NAMPT	nicotinamide phosphoribosyl transferase
NET	neutrophil extracellular traps
NGFR	nerve growth factor receptor
NLRP3	NOD-, LRR- and pyrin domain containing protein 3
NK	natural killer
PAK4	p21-activated kinase 4
PD-1	programmed cell death protein
PD-L1	programmed cell death ligand 1
PDT	photodynamic therapy
PGE2	prostaglandin E2
phospho-elF4E	phosphorylated protein eIF4E
PKA	protein kinase A
PIK3C3	phosphatidylinositol 3-kinase catalytic subunit type 3
PMN-MDSC	polymorphonuclear myeloid-derived suppressor cell
PTEN	gene-encoding phosphatase and tensin homolog deleted on chromosome 10
RAC-1	Ras-related C3 botulinum toxin substrate 1
RNLS	renalase
ROCK	Rho-associated protein kinase
ROS	reactive oxygen species
R-spondin/LGR4	R-spondin-Leucine-rich repeat containing G-protein coupled receptor 4
SGN1	Salmonella typhimurium
SK1	sphingosine kinase 1
SKCM	skin cutaneous melanoma
SOCS1	suppressor of cytokine signaling 1
SPI1	Spi-1-proto-oncogene
STAT	signal transducer and activator of transcription
TAM	tumor-associated macrophage
TCA	tricarboxylic acid
TCF4	transcription factor 4
TCR	T-cell receptor
TGF-β	transforming growth factor-beta
TIGIT	T cell immunoreceptor with Ig and ITIM domains
TIL	tumor-infiltrating lymphocyte
TIM3	T cell immunoglobulin and mucin domain-containing protein 3
TMB	tumor mutational burden
TME	tumor microenvironment
TLR	toll-like receptor
TNF	tumor necrosis factor
Treg	regulatory T cell
TREM1	triggering receptor expressed on myeloid cells
TSA	trichostatin-A
TSLP	thymic stromal lymphopoietin
VEGF	vascular endothelial growth factor
VCAM-1	vascular cell adhesion molecule 1
UCP2	uncoupling protein 2
UM	uveal melanoma
VPS34	vacuolar protein-sorting 34
ZEB1	zinc finger E-box binding homeobox 1
